# Genetic diversity and population structure of Ethiopian Sweet Sorghum [*Sorghum bicolor* (L.) Moench] germplasms using SSR markers

**DOI:** 10.1371/journal.pone.0316549

**Published:** 2025-03-06

**Authors:** Tefera Habtegiorgis Teklewold, Tsegaye Getahun Bogale, Demsachew Guadie Tseganeh, Muluken Enyew Birara, Tileye Feyissa Senbeta

**Affiliations:** 1 Department of Microbial, Cellular and Molecular Biology, College of Natural and Computational Sciences, Addis Ababa University, Addis Ababa, Ethiopia; 2 Institute of Biotechnology, Addis Ababa University, Addis Ababa, Ethiopia; 3 School of Biological Sciences, Washington State University, Pullman, Washington, United States of America; UNSTIM: Universite Nationale des Sciences Technologies Ingenierie et Mathematiques, BENIN

## Abstract

Sweet sorghum is a cereal crop in the grass family belonging to the genus *Sorghum bicolor* L. Moench. It is known in its sugary juice that is accumulated in its stalk and efficient C4 photosynthetic pathway. Only few molecular genetic diversity studies of Ethiopian sweet sorghum have been carried out. Understanding the genetic diversity of plants is the basis for genetic improvement, effective conservation and efficient utilization of genetic resources. Therefore, the objective of this study was to evaluate the genetic diversity and population structure of Ethiopian sweet sorghum genotypes collected from major growing areas of Ethiopia. In the present study, thirteen SSR markers produced a total of 136 alleles across all the 91 sweet sorghum accessions with an average of 10.46 alleles per marker. The major allele frequency per marker ranged from 0.16 to 0.41 with an average of 0.25. The number of alleles per marker ranged from 6 to 15. The mean PIC value was 0.80. The pair-wise genetic differentiation among the five studied sweet sorghum populations ranged from 0.07 to 0.19. The highest Fst (0.19) and the lowest Fst (0.07) population differentiation were observed between sweet sorghum population of South Wollo and Oromia Liyu Zone, and North Shewa and East Gojam sweet sorghum populations, respectively. The analysis of gene flow across populations showed that the highest gene flow was recorded between North Shewa and East Gojam (2.879), whereas the least gene flow was observed between South Wollo and Oromia Liyu Zone (0.618). The analysis of molecular variance revealed that 16% variation was observed among populations and 84% variation has been observed within populations. Meanwhile, the STRUCTURE and UPGMA methods of clustering suggested that the sampled sweet sorghum populations were clustered into two main groups (K =  2). This comprehensive study of genetic diversity and population structure of sweet sorghum *(Sorghum bicolor)* in Ethiopia suggests that future sweet sorghum improvement and utilization strategies should take the magnitude and pattern of genetic diversity into consideration.

## Introduction

Sweet sorghum is a cereal crop which is classified under the grass family and the genus *Sorghum bicolor* L. Moench. It is known in its sugary juice which is accumulated in its stalk and efficient C4 photosynthetic pathway [1,2]. Many researchers often considered it as “the camel crop” because of its drought tolerance along with the properties of high water and nutrient use efficiency [1–3].

The report of the previous studies indicated that, sweet sorghum is originated from North East Africa particularly from Ethiopia and Sudan [4–6]. Through time, it spreads to other African regions, Southern Asia, Europe, Australia and the United States [1]. Historical evidences show that sweet sorghum has been found in Ethiopia as early as 200 AD. Its stem has been used to chew as a snack and the grains were used for making local beer by the local people [7–9]. Even though sweet sorghum is considered as a multipurpose crop by many scientists, currently it is widely used as a syrup production and forage around the world. [1] Stated that an average yield of 1900 L of syrup per hectare can be achieved.

The main difference between sweet sorghum and grain sorghum is that; sweet sorghum has juice rich sugary stalks that grows rapidly with higher biomass [10]. As it is suggested by many scholars, *Shorghum bicolor* (L.) Moench grouped under Andropogoneae tribe, subgroup panicoideae of the grass family, Poaceae [11,12]. Furthermore the genus Sorghum is classified into five subgenera; these are: Sorghum, Stipo sorghum, Chaetosorghum, Heterosorghum, and Parasorghum [12]. The subgenus Sorghum contains three species including *S. bicolor, S. propinquum*, and *S. halepense* [12]. In addition, *Sorghum bicolor* has three subspecies including *Sorghum bicolor*, *Sorghum bicolor* drummondii, and *Sorghum bicolor* verticilliforum [11–14].

Based on morphological features such as grain shape, glume, and panicle, cultivated varieties of *Sorghum bicolor* have been classified into five basic races including bicolor, guinea, caudatum, kafir, and durra [12,13]. Sweet sorghum and grain sorghum have different class of race. Most of the grain sorghum varieties belong to the races caudatum, kafir, and durra, whereas sweet sorghum and forage sorghum varieties are mainly grouped in the race bicolor [12,15,16]. On the contrary, later studies showed that sweet sorghum has a polyphyletic origin and therefore, apart from race bicolor, may have parentage from other previously mentioned races as well [12,17]. Other studies suggested that in the continent of Africa particularly in Ethiopia where most of the wild germplasm has originated, there are intermediate varieties of *sorghum bicolor* [12,18].

Understanding the genetic variation of plants has great importance in crop improvement program [19]. Genetic improvement of a crop is mainly dependent on the strength of genetic diversity within the crop species. Furthermore, understanding the molecular basis of the essential biological phenomena in plants is important for the effective conservation, management, and efficient utilization of plant genetic resources.

Molecular markers are nucleotide sequence found in the genome of living organisms and their variations between accessions enable to identify the pattern of inheritance [20]. Molecular markers help breeders to select suitable genotypes for crossing in breeding programs [19]. Evaluation of genetic diversity can be studied using different ways, among the most efficient of which is molecular markers [20]. Simple sequence repeats (SSR) markers are characterized by their tandem repeats which range one to six nucleotides. They are distributed throughout the genome of an organism, and have multiple alleles that often have the same loci between related species [19–21]. They have been widely used in different genetic studies such as diversity analysis, quantitative trait locus mapping, and genotype identification [19].

Even though the Ethiopian Biodiversity Institute contains large number of sorghum germplasms in its gene bank, most of the collections are not well characterized. In addition sweet sorghum germplasms are not maintained separately from grain sorghum germplasms [22]. Furthermore, only a few molecular studies of Ethiopian sweet sorghum have been carried out to show the extent of genetic variation. However, these studies did not include germplasms of sweet sorghum from all geographical range of Ethiopia. Therefore, the objective of this study was to evaluate the genetic diversity and population structure of Ethiopian sweet sorghum genotypes collected from major growing areas of Ethiopia particularly those areas which were not covered in the previous studies.

## Materials and methods

### Plant materials

**Fig 1:**
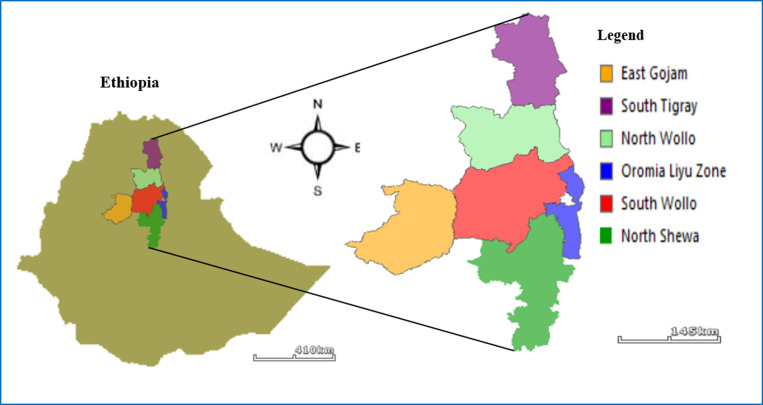
Collection sites of the studied sweet sorghum accessions.

In this study, a total of ninety one sweet sorghum accessions were used. From these accessions, twenty nine of them were obtained from Ethiopian Biodiversity Institute (EBI) and sixty two of them were collected from major sweet sorghum growing areas of Amhara and Tigray regions of Ethiopia (Fig 1). Those sweet sorghum accessions that were obtained from Ethiopian Biodiversity Institute (EBI) were a mixture of grain sorghum; therefore, purification and isolation have been done by cultivating them in the farm during 2022 growing season. While they reach at maturity stage (3 to 4 months), they possessed distinctive morphological features that make them different from grain sorghum. The color of the midrib of their leaf and the juice accumulated in their stalk were used to differentiate them from grain sorghum. The remaining sixty two sweet sorghum accessions were collected directly from farmer’s sweet sorghum farms during 2022 harvesting season. Ethiopian farmers cultivate a mixture of sweet sorghum and grain sorghum on the same plot of land at the same time. They use the sweet sorghum juicy stalk as a chewing snack and the grain sorghum for making foods such as local pancake called ‘*Injera’* and local beverage. Farmers from the selected areas of Amhara and Tigray regions, were the sources for the collection of these sixty two sweet sorghum accessions. Based on their collection sites, they were categorized into five populations: North Shewa, South Tigray, South Wollo, East Gojam and Oromia Liyu Zone.

### DNA extraction

All the ninety one sweet sorghum accessions were grown in a pot at the greenhouse of Institute of Biotechnology, Addis Ababa University. Healthy young leaves were collected from two-week-old plants of each accession and kept in plastic bag containing silica gel. Genomic DNA was extracted from young leaves using modified CTAB plant DNA extraction protocol [23]. DNA quality was checked by loading 2µl of DNA mixed with 2µl of loading dye with 4µl of ethidium bromide on a 1% agarose gel and separated at 100 V for 45 minutes. The extracted genomic DNA was quantified using a Nano-Drop spectrophotometer (ND-2000) and stored at − 20°C until used for further analyses.

### PCR and gel electrophoresis

Twenty SSR primer-pairs developed by [24] were used for initial screening, amplification, polymorphism and specificity to target loci. It could have been more appropriate to use large number of SSR markers in this study; however due to limitation of resources such as chemicals and reagents for running polymerase chain reaction and gel electrophoresis, limited number of markers were used. According to the manufacturer’s instruction, the primers were dissolved using nuclease-free water to a final concentration of 100 mol/µl and further diluted in 1 to 10 ratio. Out of the twenty screened primer pairs, thirteen of them amplified the genomic DNA and showed polymorphism across tested sweet sorghum accessions (Table 1). DNA amplification was performed in 11μl reaction volume containing 4μl of 2X Taq plus Master Mix (containing Taq DNA polymerase, dNTPs, MgCl_2_, PCR buffer, PCR reaction enhancer, stabilizer and blue tracer dye) (Sangon Biotech), 1μl of 100 ng/µl template DNA, 0.5μl forward and 0.5µl reverse primers, and 5µl of nuclease free water. The PCR program for the primer pairs consisted of 4 min at 94°C for the initial denaturation followed by 35 cycles of 94°C for 30 second, primer annealing at 52-60°C for 30 seconds, extension cycle at 72°C for 1min followed by final extension at 72°C for 10min and then hold at 4°C. The PCR amplified products were separated by loading 10µl of each of the PCR product on 2% agarose gel electrophoresis which is mixed with 4 µl of ethidium bromide and run using 1 × TBE buffer at 100 V for 2hours. The product was finally visualized under gel documentation system (BioDoc-ItTM imaging system) and subsequently photographed. A 100 bp size molecular marker (DNA ladder) was used to estimate the size of the amplified products.

**Table 1:** List of SSR markers used to amplify the genomic DNA of sweet sorghum.

No.	Primer’s name	Repeat motif	Chromosome number	Forward primer	Reverse primer	Annealing Temperature
1	Xcup53	(TTTA)5	1	GCAGGAGTATAGGCAGAGGC	CGACATGACAAGCTCAAACG	56
2	Xtxp015	(TC)16	5	CACAAACACTAGTGCCTTATC	CATAGACACCTAGGCCATC	54
3	Xisep0310	(CCAAT)4	2	TGCCTTGTGCCTTGTTTATCT	GGATCGATGCCTATCTCGTC	57
4	Xtxp114	(AGG)8	3	CGTCTTCTACCGCGTCCT	CATAATCCCACTCAACAATCC	57
5	Xtxp012	(CT)22	4	AGATCTGGCGGCAACG	AGTCACCCATCGATCATC	57
6	Xtxp273	(TTG)20	8	GTACCCATTTAAATTGTTTGCAGTAG	CAGAGGAGGAGGAAGAGAAGG	*56*
7	Xtxp321	(GT)4+(AT)6+(CT)21	8	TAACCCAAGCCTGAGCATAAGA	CCCATTCACACATGAGACGAG	60
8	Xtxp265	(GAA)19	6	GTCTACAGGCGTGCAAATAAAA	TTACCATGCTACCCCTAAAAGTGG	*56*
9	SbAGE01	(AG)30	2	GACCGATCTAATGATGCAG	ACGGTAGAGAAGACCCATC	54
10	Xtxp21	(AG)18	4	GAGCTGCCATAGATTTGGTCG	ACCTCGTCCCACCTTTGT TG	*52*
11	Xtxp12	(CT)22	4	AGATCTGGCGGCAACG	AGTCACCCATCGATCATC	56
12	Xtxp3	(CT)8+(CT)36	2	AGCAGGCGTTTATGGAAG	ATCCTCATACTGCAGGAC C	57
13	Xtxp43	(CT)28	1	AGTCACAGCACACTGCTTGTC	AATTTACCTGGCGCTCTG C	60

### Data analysis

**Fig 2:**
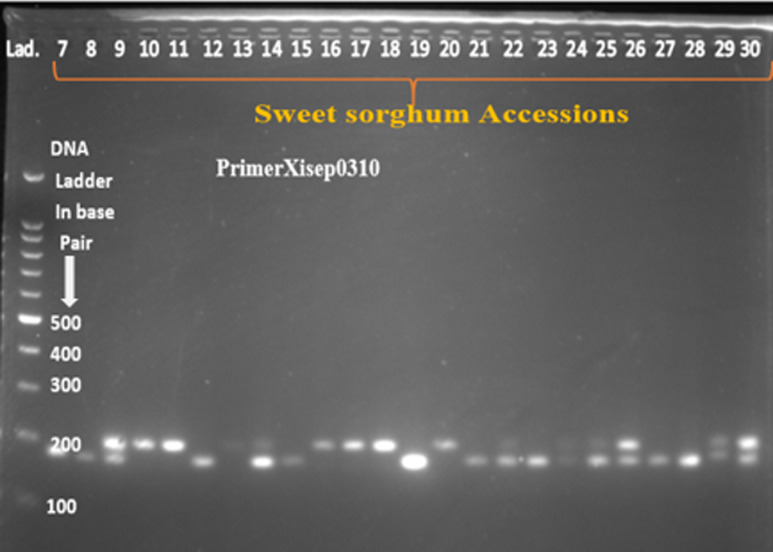
The band pattern of some of the sweet sorghum accessions amplified by primer Xisep0310.

**Fig 3:**
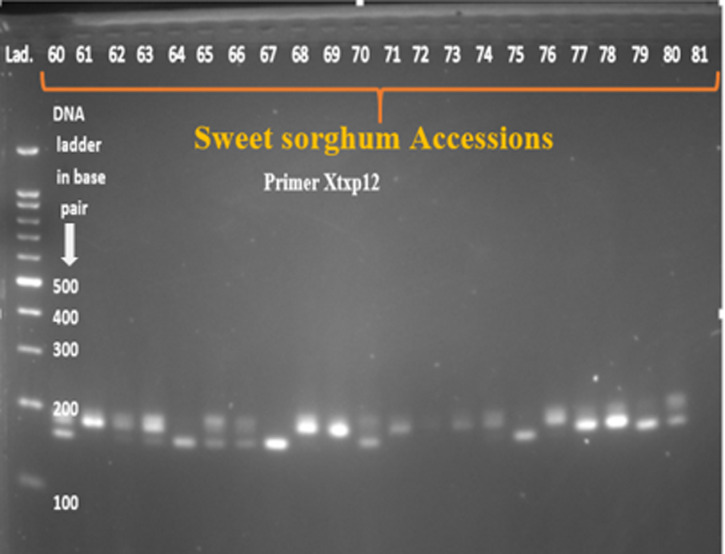
The band pattern of some of the sweet sorghum accessions amplified by primer Xtxp12.

**Fig 4:**
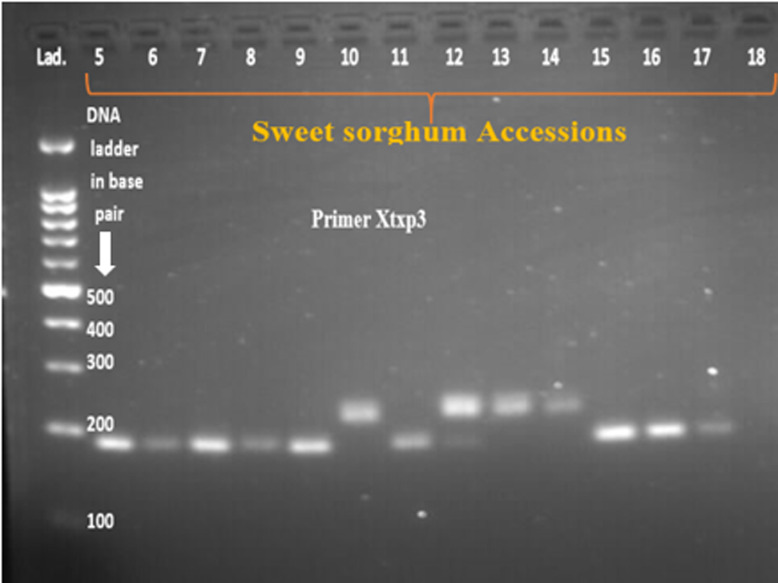
The band pattern of some of the sweet sorghum accessions amplified by primer Xtxp3.

**Fig 5:**
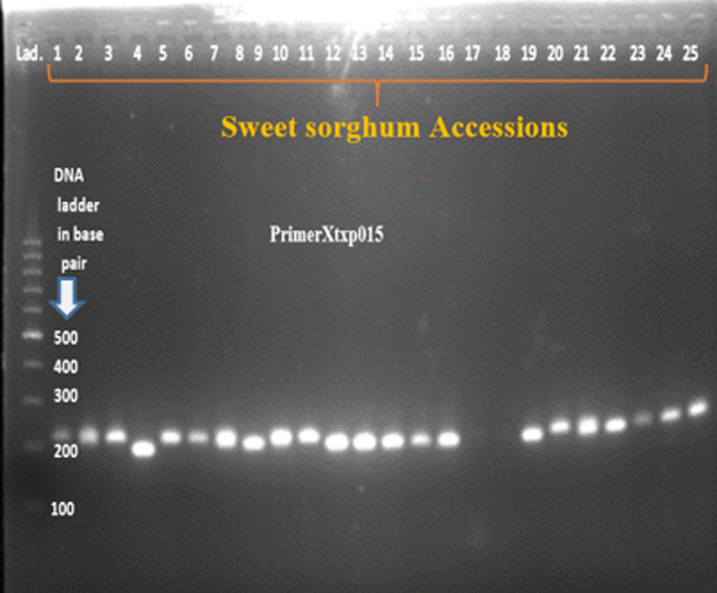
The band pattern of some of the sweet sorghum accessions amplified by primer Xtxp015.

After PCR amplification and gel electrophoresis have been conducted, the bands were photographed by BioDoc-ItTM imaging system (Figs 2–5). All of the bands of the studied sweet sorghum accessions under different SSR markers were scored using Image Lab™ Touch software version 2.3. All the molecular analysis were carried out based on the molecular weight of each bands. Parameters used to characterize and analyze the SSR markers such as major allele frequency (MAF), the number of allele (Na), gene diversity (GD) and Polymorphic information contents (PIC) were analyzed using Power marker version 3.25 software [25]. On the other hand, number of different alleles, effective number of alleles, Shannon’s information index, number of private alleles, expected heterozygosity, observed heterozygosity, fixation index, pairwise population genetic distance, genetic differentiation, Principal Coordinate Analysis (PCOA) and molecular variance (AMOVA) were analyzed using GenAlEx version 6.503 software [26]. The two integrated, STRUCTURE version 2.3.4 and Structure harvester software were used to estimate population structure. The structure 2.3.4 software was used to estimate the optimum number of clusters (K) of the studied sweet sorghum accessions. The software uses Bayesian algorithm and admixture model based on Monte Carlo Markov Chain algorithm [27]. In the sweet sorghum population structure estimation, the admixture model used the allele frequency correlation value. The number of ancestral sweet sorghum populations was calculated for K ranging from 1 to 8 with 8 runs for each K value. For each run, a burn-in period of 30000 iterations was used. In addition, MCMC (Markov Chain Monte Carlo) replications of 15000 was performed. The structure calculation results were submitted to the online tool Structure Harvester [28] to determine the number of clusters in the group [29]. Furthermore, cluster analysis and phylogenetic tree construction were carried out by the combination of MEGA version 11.0.13 [30] and power marker version 3.25 [25] software.

## Results

### Polymorphism and allelic diversity

Thirteen SSR markers produced a total of 136 alleles across all 91 sweet sorghum accessions with an average of 10.46 alleles per marker. The major allele frequency per marker ranged from 0.16 (Xtxp012) to 0.41 (Xtxp114), with an average of 0.25. The number of alleles per marker ranged from 6 (Xtxp114) to 15 (Xtxp12) (Table 2). All of the SSR markers were highly polymorphic. The PIC values for the SSR markers ranged from 0.68 to 0.88 with an average of 0.80 (Table 2). The highest PIC value of 0.88 was obtained for marker Xtxp012, whereas the lowest PIC value was recorded for marker Xtxp114. The overall heterozygosity (Ho) over loci varied from 0.00 (Xcup53, Xtxp015, Xtxp114 and SbAGE01) to 0.26 (Xtxp12) with an average of 0.12. The gene diversity index ranged from 0.72 (Xtxp114) to 0.89 (Xtxp012).

**Table 2:** Patterns of alleles amplified by 13 SSR markers.

Markers	Major Allele Frequency	Sample Size	Allele No	PIC	observed Heterozygosity	Gene Diversity
Xcup53	0.19	91	13	0.85	0.00	0.87
Xtxp015	0.23	91	11	0.82	0.00	0.84
Xisep0310	0.22	91	8	0.80	0.40	0.83
Xtxp114	0.41	91	6	0.68	0.00	0.72
Xtxp012	0.16	91	15	0.88	0.19	0.89
Xtxp273	0.30	91	11	0.78	0.10	0.81
Xtxp321	0.36	91	10	0.71	0.24	0.75
Xtxp265	0.18	91	11	0.86	0.15	0.87
SbAGE01	0.19	91	11	0.84	0.00	0.86
Xtxp21	0.21	91	8	0.82	0.01	0.84
Xtxp12	0.25	91	9	0.79	0.26	0.82
Xtxp3	0.32	91	9	0.76	0.02	0.79
Xtxp43	0.21	91	14	0.83	0.16	0.84
Mean	0.25	91	10.46	0.80	0.12	0.82
Maximum value	0.41	–	15	0.88	0.40	0.89
Minimum value	0.16	–	6	0.68	0.00	0.72

The total expected heterozygosity (Ht) recorded for all 13 SSR markers in the present study ranged from 0.71 to 0.88 with a mean of 0.82 (Table 3). The mean value of expected heterozygosity (He) revealed by each SSR markers ranged from 0.54 (Xtxp114) to 0.79 (Xtxp012) (Table 3). On the other hand, the mean value of the observed heterozygosity (Ho) ranged from 0.00 (Xcup53, Xtxp015, Xtxp114, SbAGE01) to 0.35 (Xisep0310 and Xisep0310) (Table 3). The overall mean of expected heterozygosity (He) and observed heterozygosity (Ho) of the 13 SSR markers were 0.66 and 0.14, respectively (Table 3).

**Table 3:** F-statistics and estimates of gene flow (Nm) over all populations for each markers.

Markers	Ht	Mean He	Mean Ho	Fis	Fit	Fst	Nm
Xcup53	0.86	0.66	0.00	1.00	1.00	0.22	0.84
Xtxp015	0.83	0.67	0.00	1.00	1.00	0.19	1.05
Xisep0310	0.83	0.69	0.35	0.48	0.57	0.16	1.28
Xtxp114	0.71	0.54	0.00	1.00	1.00	0.24	0.77
Xtxp012	0.88	0.79	0.21	0.73	0.76	0.10	2.17
Xtxp273	0.83	0.62	0.14	0.77	0.80	0.25	0.74
Xtxp321	0.78	0.64	0.34	0.46	0.56	0.17	1.14
Xtxp265	0.85	0.67	0.11	0.83	0.86	0.21	0.93
SbAGE01	0.82	0.71	0.00	1.00	1.00	0.13	1.61
Xtxp21	0.82	0.61	0.04	0.93	0.95	0.26	0.70
Xtxp12	0.81	0.73	0.35	0.51	0.55	0.09	2.29
Xtxp3	0.81	0.58	0.04	0.91	0.93	0.28	0.63
Xtxp43	0.85	0.66	0.19	0.70	0.77	0.22	0.84
Mean	0.82	0.66	0.14	0.79	0.83	0.19	1.15
Maximum value	0.88	0.79	0.35	1.00	1.00	0.28	2.29
Minimum value	0.71	0.54	0.00	0.46	0.55	0.09	0.63

Key: Ho =  Observed Heterozygosity =  No. of Hets/ N, He =  Expected Heterozygosity =  1 - Sum pi^2, Fis =  (Mean He - Mean Ho)/ Mean He, Fit =  (Ht - Mean Ho)/ Ht, Fst =  (Ht - Mean He)/ Ht, Nm =  [(1/ Fst) - 1]/ 4, Mean He =  Average He across the populations. Mean Ho =  Average Ho across the populations. Ht =  Total Expected Heterozygosity =  1 - Sum tpi^2 where tpi is the frequency of the i^th^ allele for the total & Sum tpi^2 is the sum of the squared total allele frequencies. Note: Mean F-Statistics represent arithmetic averages.

### Genetic diversity within and among populations

Among the studied sweet sorghum populations, sweet sorghum population of North Shewa revealed high mean value in terms of number of different alleles (8.46), number of effective alleles (5.15), expected heterozygosity (0.79), Shannon’s Information Index (1.79) and unbiased expected heterozygosity (0.80) (Table 4). On the contrary, sweet sorghum populations of South Wollo showed the least number of different alleles (3.462). In terms of other genetic diversity parameters, Oromia Liyu Zone revealed the least number of effective alleles (2.500), expected heterozygosity (0.541), observed heterozygosity (0.006), Shannon’s Information Index (0.994), unbiased heterozygosity (0.564) and fixation index (0.989) (Table 4). North Shewa population revealed the highest mean value of private alleles (1.54), whereas the least number of private alleles were recorded in the sweet sorghum population of Oromia Liyu Zone and East Gojam (0.77). The analysis of the number of different alleles with a frequency>= 5%, revealed that relatively high mean value (5.69) was scored for North Shewa population and the least mean value was scored in South Wollo population (3.462). On the other hand, the number of locally common alleles with frequency>= 5% showed that North Shewa population has revealed the highest mean value (2.00), whereas South Wollo population has shown the least mean value (0.46) (Table 4).

**Table 4:** Mean allelic patterns across populations.

	Na	Na Freq.>= 5%	Ne	I	No. Private Alleles	No. LComm Alleles (<=25%)	No. LComm Alleles (<=50%)	Ho	He	uHe	F
South Wollo	3.462	3.462	2.862	1.084	0.308	0.000	0.462	0.110	0.617	0.664	0.840
South Tigray	5.923	4.308	3.872	1.408	0.615	0.000	1.385	0.105	0.681	0.697	0.823
Oromia Liyu Zone	3.692	3.615	2.500	0.994	0.077	0.000	0.538	0.006	0.541	0.564	0.989
North Shewa	8.462	5.692	5.146	1.793	1.538	0.000	2.000	0.137	0.791	0.800	0.832
East Gojam	4.077	4.077	3.370	1.265	0.077	0.000	1.231	0.338	0.683	0.759	0.539
Maximum value	8.462	5.692	5.146	1.793	1.538	0.000	2.000	0.338	0.791	0.800	0.989
Minimum value	3.462	3.462	2.500	0.994	0.077	0.000	0.462	0.006	0.541	0.564	0.539

Key: Na =  No. of Different Alleles, Na (Freq>= 5%) =  No. of Different Alleles with a Frequency>= 5%, Ne =  No. of Effective Alleles =  1/ (Sum pi^2), I =  Shannon’s Information Index =  -1 * Sum (pi *  Ln (pi)), No. Private Alleles =  No. of Alleles Unique to a Single Population, No. LComm Alleles (<=25%) =  No. of Locally Common Alleles (Freq.>= 5%) Found in 25% or Fewer Populations, No. LComm Alleles (<=50%) =  No. of Locally Common Alleles (Freq.>= 5%) Found in 50% or Fewer Populations, He =  Expected Heterozygosity =  1 - Sum pi^2, uHe =  Unbiased Expected Heterozygosity =  (2N/ (2N-1)) *  He, F =  Fixation Index =  (He - Ho)/ He =  1 - (Ho/He), Where pi is the frequency of the i^th^ allele for the population & Sum pi^2 is the sum of the squared, population allele frequencies.

### Genetic differentiation, genetic distance and gene flow

The pair-wise genetic differentiation among the populations ranged from 0.071 to 0.196 (Table 5). The highest population differentiation was observed between South Wollo and Oromia Liyu Zone (Fst =  0.196) followed by East Gojam and Oromia Liyu Zone (Fst =  0.186). The lowest population differentiation (Fst =  0.071) was observed between North Shewa and East Gojam (Table 5). The highest Nei’s genetic distance was observed between East Gojam and Oromia Liyu Zone (1.201) followed by East Gojam and South Tigray (1.080). The lowest Nei’s genetic distance (0.543) was observed between East Gojam and North Shewa (Table 5). The highest gene flow across populations was recorded between North Shewa and East Gojam (2.879), whereas the least gene flow was observed between South Wollo and Oromia Liyu Zone (0.618) (Table 6).

**Table 5:** Pairwise population genetic differentiation analysis measured by Fst (below the diagonal) and Nei Genetic Distance (above the diagonal) among different geographic regions.

	South Wollo	South Tigray	Oromia Liyu Zone	North Shewa	East Gojam
South Wollo	0.000	0.949	1.067	0.860	0.972
South Tigray	0.144	0.000	0.819	0.757	1.080
Oromia Liyu Zone	0.196	0.159	0.000	0.769	1.201
North Shewa	0.111	0.091	0.128	0.000	0.543
East Gojam	0.145	0.134	0.186	0.071	0.000

**Table 6:** Pairwise population gene flow (Nm) Values (below diagonal).

	South Wollo	South Tigray	Oromia Liyu Zone	North Shewa	East Gojam
South Wollo	0.000				
South Tigray	0.939	0.000			
Oromia Liyu Zone	0.618	0.815	0.000		
North Shewa	1.359	1.474	1.101	0.000	
East Gojam	1.114	1.061	0.675	2.879	0.000

### Analysis of molecular variance

Analysis of molecular variance (AMOVA) revealed that majority of the variance was restricted within populations (84%), whereas 16% variance was partitioned among populations (Table 7).

**Table 7:** Analysis of molecular variance (AMOVA) among and within sweet sorghum populations based on genetic distance matrix.

Source	df	SS	MS	Est. Var.	Percentage of variation
Among Pops	4	278.399	69.600	3.387	16%
Within Pops	86	1541.710	17.927	17.927	84%
Total	90	1820.110		21.313	100%

### Cluster analysis

**Fig 6:**
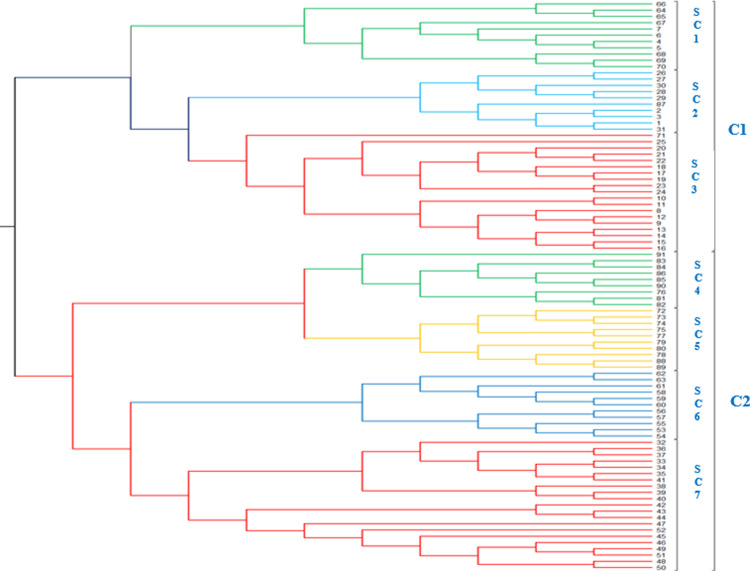
Unweighted pair-group method with arithmetic mean cluster analysis showing the genetic relationship among the 91 sweet sorghum accessions using 13 SSR markers.

The phylogenetic analysis revealed clustering of sweet sorghum accessions is mainly based on their geographical origin with a few exceptions (Fig 6). The 91 accessions grouped into two major clusters. The first cluster (C1) consist of accessions from South Wollo and South Tigray Zones. The second cluster (C2) consisted of accessions collected from the North Shewa, East Gojam, and Oromia liyu Zone (Fig 3). The phylogenetic tree also showed that the two major clusters further grouped into seven sub clusters (SC1 to SC7). Three sub clusters and forty accessions were included in the first cluster. The remaining four sub clusters with fifty one accessions were included in the second cluster. Some accessions collected from North Shewa were clustered along with accessions collected from South Wollo.

### Principal coordinates analysis

**Fig 7:**
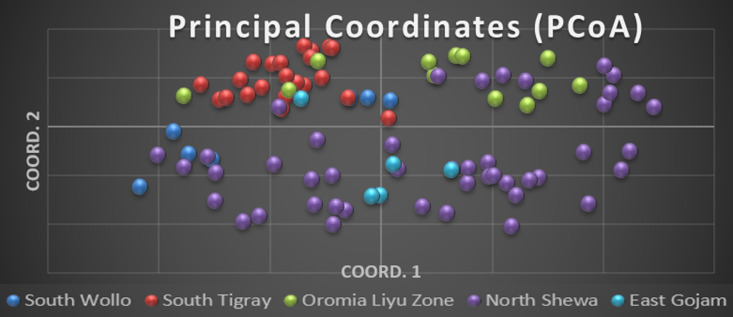
Principal coordinates analysis of 91 sweet sorghum accessions using 13 SSR markers.

The Principal coordinate analysis (PCoA) result revealed 31.26% of variations explained by the first three axes. The first, second and third principal coordinate axes showed 12.55%, 10.38% and 8.33%, respectively (Table 8). Although the groups were not completely separated in the two-dimensional plot, the distribution of accessions on the plot indicated the presence of genetic diversity (Fig 7). Most of the accessions collected from North Shewa zone were clustered on the lower left and right sides of the coordinate graph. In addition, some sweet sorghum accessions are concentrated on the upper right side of the coordinate graph along with the accessions collected from Oromia Liyu Zone. On the other hand, the accessions of South Tigray were clustered on the upper left and most of the accessions of Oromia Liyu Zone were concentrated on the upper right of the axis. All accessions collected from North Shewa, South Wollo and East Gojam zones aggregated together on the lower left and right sides of the coordinate graph and there is no clear separation among them.

**Table 8:** Percentage of variation explained by the first 3 axes.

Axis	1	2	3
%	12.55	10.38	8.33
Cumulative %	12.55	22.93	31.26

### Population structure

**Fig 8:**
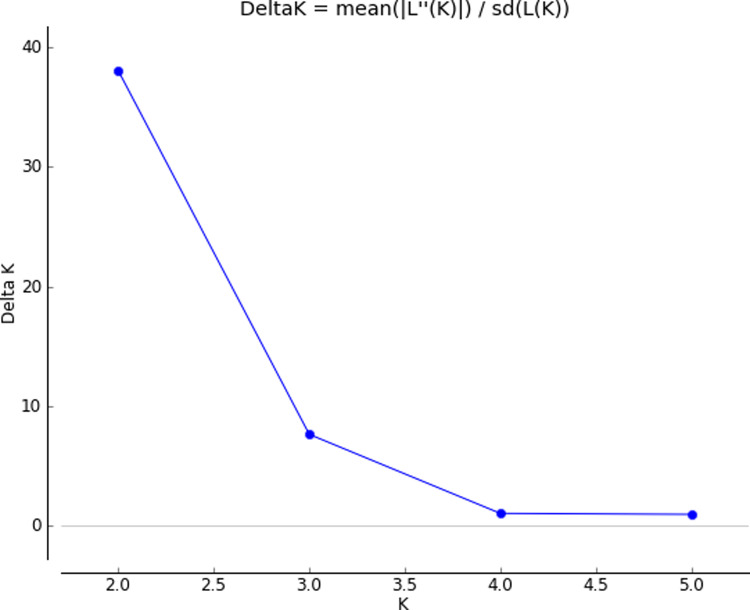
Bayesian model-based estimation of population structure (K=  2) of the 91 sweet sorghum accessions.

**Fig 9:**
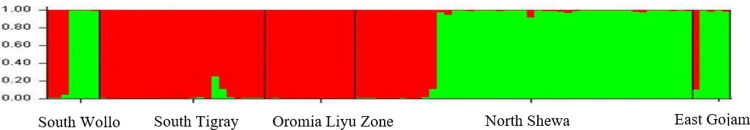
Bar Plot showing the Population structure of 91 sweet sorghum accessions using 13 SSR molecular markers with (K=  2).

In this study the population structure results for the sweet sorghum populations obtained using STRUCTURE software showed that the maximum value for ‘K’ is 2 (Fig 8). The population structure analysis indicated that the 91 accessions were optimally divided into 2 groups. The genetic structure of sweet sorghum confirmed that the pattern of population structure is closely related to the geographical distribution of accessions. Accessions from adjacent area were grouped together (Fig 9). Although there was some degree of genetic mixing (gene exchange) between groups, there was still clear differentiation. The results of PCoA and unweighted pair-group method with arithmetic mean (UPGMA) cluster analysis further supported those from STRUCTURE software. Individuals from the adjacent area tended to cluster together.

## Discussion

### Polymorphism of SSR markers and allelic patterns

Allele frequency is the concept used to quantify genetic variation. In this study, thirteen SSR markers produced a total of 136 alleles across all 91 sweet sorghum accessions with an average of 10.46 alleles per marker. In a similar study, [22] obtained a total of 159 alleles using 13 markers in 202 sweet sorghum accessions. In addition, [31] in his study, reported a total of 123 alleles with an average of 10.25 alleles per marker and the major allele frequency per marker ranged from 0.159 (Xtxp012) to 0.41 (Xtxp114), with an average of 0.25. The study conducted by [32] reported the average major allele frequency of 0.5, which is higher than 0.25. In the present study higher numbers of alleles were recorded in comparison with the previous studies. This difference could be due to the types of markers, the size of the population, and the number of germplasms. This variation of alleles and genotypes present in the populations can be a basis for selection and breeding improvement programs.

Polymorphic information content (PIC) is the ability of a marker to establish polymorphism in the population based on the number of alleles discovered and on their distribution frequency [33]. Having a high PIC value for a marker is one of the most important indicators that the marker can be used successfully in the evaluation of genetic variation [34]. The study conducted on Ethiopian sweet sorghum by [22], the PIC of SSR markers values ranged from 0.37 to 0.85. Another study conducted on sorghum by [31], the range of PIC value was 0.39 to 0.85 using 12 SSRs across 160 accessions. The study of [19] also recorded a PIC value of 0.22 to 0.75 with a mean value of 0.52. Furthermore, the study conducted by [32] reported an average PIC value of 0.67. In this study relatively higher PIC values were observed compared to the previous studies. The PIC values for the 13 SSR primers ranged from 0.68 to 0.88 with an average value of 0.80. According to [35,36], markers with a PIC value > 0.5 are considered to be highly informative, 0.25 to 0.5 moderately informative and less than 0.25 slightly informative. In fact, PIC is determined by heterozygosity and number of alleles [37]. In this study the mean PIC value of 0.80 confirmed that the markers used were highly informative. This implies that the SSR markers used in this study are effective in showing the diversity. Therefore, these SSR markers can be a choice for future genetic characterization and diversity studies.

The average number of private alleles observed in this study ranged from 0.08 (East Gojam and Oromia Liyu Zone) to 1.538 (North Shewa). According to [33], expected hetrozyousity (He) can be defined as the likelihood that an individual in the population is heterozygous for the locus. Observed heterozygosity (Ho) is the fraction of heterozygous genes in a population. The total expected heterozygosity recorded for all 13 SSR markers in the present study ranged from 0.71 (Xtxp114) to 0.88 (Xtxp012) with a mean of 0.82. In this study, the observed heterozygosity (Ho) ranged from 0.00 (Xcup53, Xtxp015, Xtxp114 and SbAGE01) to 0.35 (Xisep0310) with a mean of 0.14. This result is higher than the He value reported by [31] which was 0.66 and that reported by [22], in which the expected heterozygosity (He) ranged from 0.42 to 0.85 with a mean of 0.72 and the overall observed heterozygosity (Ho) ranged from 0.04 to 0.16 with an average of 0.09. This difference could be because of the difference in SSR markers used, the size of the studied population and the number of accession used in the study. Among the studied sweet sorghum populations, North Shewa population revealed high mean value of number of different alleles (8.46), number of effective alleles (5.15), expected heterozygosity (0.79), observed heterozygosity (0.14), unbiased expected heterozygosity (0.80) and Shannon’s Information Index (1.79) and fixation index (0.83).

The gene diversity (GD) of a locus, also known as its expected heterozygosity (He) describes the expected proportion of heterozygous genotypes under Hardy-Weinberg Equilibrium [38]. The high levels of heterozygosity that was recorded in this study implies the presence of substantial genetic diversity that breeders can explore. Allelic patterns across these five populations of sweet sorghum showed the presence of private alleles and implication for breeding is that the private alleles are an indication of new genes that could be crucial in desired or novel trait. On the contrary, sweet sorghum populations of Oromia Liyu Zone revealed the least mean value of number of effective alleles (2.50), expected heterozygosity (0.54), Shannon’s Information Index (0.99), observed heterozygosity (0.01), unbiased expected heterozygosity (0.56), and fixation index (0.99). All populations exhibited unique private alleles. The highest number of private alleles (1.54) were exhibited by North Shewa populations and the least number of private alleles (0.08) was displayed by East Gojam and Oromia Liyu Zone populations. The private allelic patterns across the sweet sorghum populations suggest the presence of new genes that may be contributing to desired traits.

### Genetic differentiation and population structure

Genetic differentiation (Fst) measures the amount of genetic variance that can be explained by population structure based on Wright’s F-statistics. [39] Suggested that genetic differentiation among populations can be categorized as (Fst < 0.05, low; 0.05_Fst < 0.15, medium; Fst > 0.15, high). In the present study, 0.19 average value of Fst was recorded. The highest population differentiation was observed between South Wollo and Oromia Liyu Zone populations (Fst =  0.19), followed by East Gojam and Oromia Liyu Zone (Fst =  0.18). The lowest population differentiation (Fst =  0.07) was observed between North Shewa and East Gojam populations. In this study, the pair wise Fst value varied from 0.07 to 0.19. This analysis showed that there was high level of genetic differentiation between each pair of populations. Since all the results of Fst so far observed in this study were very low and approximated to 0, it showed that they are not completely isolated one another and there is sharing of genetic material through breeding activity among the studied sweet sorghum population.

The pairwise Nei’s genetic distance analysis showed that the lowest (0.54) and the highest (1.20) Nei’s genetic distance were recorded among the studied populations. The highest Nei’s genetic distance was observed between East Gojam and Oromia Liyu Zone (1.20) populations followed by East Gojam and South Tigray (1.08). The lowest Nei’s genetic distance (0.54) was observed between East Gojam and North Shewa. East Gojam population showed the highest (1.20) pairwise Nei’s genetic distance with South Tigray and Oromia Liyu Zone populations and is the most genetically distinct population. This can be partly explained by the fact that the location of East Gojam is geographically the most distant to Oromia Liyu Zone and South Tigray. This may restricted the exchange of seeds among farmers, hence, these populations may serve as potential sources of new genetic variation of important traits that can be used in sweet sorghum breeding programs.

According to [39], the gene flow (Nm) is divided into three grades: high (≥1.0), medium (0.250–0.99) and low (0.0–0.249). When Nm >  1 existed, there is certain gene flow between populations, in the present study, 1.15 average value of Nm was recorded. This result suggest that there was high gene flow among the studied sweet sorghum populations. The analysis of gene flow showed the highest gene flow was recorded between North Shewa and East Gojam (2.879) populations whereas the least gene flow was observed between South Wollo and Oromia Liyu Zone (0.618) populations.

AMOVA revealed that the variance within populations is higher than that of the variance observed among populations. The majority of the variance was within populations (84%), whereas 16% variance was among populations. This significant differences between accessions might be due to high sexual recombination or the mutation of a number of repeats of a given genotype. Additionally, this high genetic variation could be due to the natural selection process. On the contrary, the relatively low variation observed among population could be the result of gene flow through exchange of seeds via breeding programs or market. Since the variation present among population is lower than within population, it may implies the influence of climatic factor is low in the expression of the genetic diversity of accessions. Similar study conducted by [40], revealed that 68.1% of variation was recorded within population and 31.9% was restricted among the studied sorghum populations. The study conducted on grain sorghum by [41] revealed that 93.26% of the total genetic variation within populations and 6.74% among populations. Another study conducted by [31] on grain sorghum using 12 SSR markers showed that 54.44% of the variation was observed within populations and 32.76% was among populations. Furthermore, the study conducted by [42] on 200 accessions of grain sorghum using 39 SSR markers reported that 99.62% of the variation was accounted to within sweet sorghum populations and 0.38% to the total variability observed was among the studied sweet sorghum populations.

Genetic differentiations and relationships observed among sweet sorghum accessions using distance methods, pairwise differentiation, PCoA, UPGMA analysis and Bayesian model-based cluster analysis confirmed the differentiation of accessions based on their geographical location although there are considerable intermixes. As the cluster analysis of this study revealed, most of the sweet sorghum accessions collected from the same geographical areas were clustered together. The unweighted pair-group method with arithmetic mean (UPGMA) grouped the 91 sweet sorghum accessions into two broad classes. This clustering of sweet sorghum accessions showed that there was significant relationship between geographic origin and genetic similarity, although there is considerable intermixes. On the other hand, some sweet sorghum accessions that were collected from areas that are not adjacent to each other clustered under the same group. This clustering suggests that there were some level of gene flow and exchange of seeds among sweet sorghum growing areas in Ethiopia through market or breeding programs. This pattern of genetic relationships where accessions from the same or adjacent zones were genetically related could be attributed to existence of variety exchange patterns of such accessions among farmers.

PCoA is a multivariate dataset that provides the ability to find and archive key patterns in multiple loci and multiple samples [43]. PCoA can be utilized to derive a 2 or 3-dimensional scatter plot of individuals, such that the geometrical distances among individuals in the plot reflect the genetic distances among them with minimal distortion. Aggregations of individuals in such a plot will reveal sets of genetically similar individuals [43–45]. PCoA results revealed similarities of accessions from North Shewa, South Wollo and East Gojam. On the other hand accessions of South Tigray and Oromia Liyu Zone showed similarity. This similarity could be because of gene flow among farmers. As it is shown by the PCoA analysis, the North Shewa populations shared with other populations. On the contrary, South Tigray population was uniquely isolated from the other populations indicating absence of exchange of genetic material. The overall distribution of the accessions on the diagram showed that there is a gene flow process among sweet sorghum accessions especially in areas that are geographically adjacent to each other. The distribution also shows that genetic diversity is quite strong among sweet sorghum populations collected from different areas of Ethiopia. According to [40,46], several factors could have contributed to the detected patterns of genetic diversity and observed structure. These include environmental, biological, cultural, and socioeconomic factors that play a role in farmer’s decisions to choose or keep a particular sweet sorghum cultivars at any given time.

## Conclusions

Unlocking the genetic diversity of germplasm is the first step for improvement and development of superior cultivars for different breeding purpose. The present study reports the molecular genetic diversity studies and population structure analysis of Ethiopian sweet sorghum accessions collected from different parts of Ethiopia using SSR markers. The SSR markers employed in this study exhibited a high degree of polymorphism, effectively revealing the genetic differences among and within the sweet sorghum populations. The high informative degree of these SSR markers make them valuable candidates for future genetics studies. The results of this study showed a higher level of genetic diversity within the studied sweet sorghum populations than between sweet sorghum populations. This indicates the presence of gene flow among the sweet sorghum accessions sharing close geographical distance and similar ecology. This high level of genetic variability will have a great role for future genetic improvement through breeding. It also contributes a lot for conservation of the sweet sorghum accessions in their native habitat.

The present study identified the sweet sorghum accessions collected from different agroecologies of Ethiopia were classified into two main groups. Although there is considerable intermixes, this clustering of sweet sorghum accessions showed significant relationship between geographic origin and genetic similarity. Additionaly, the study identified the role of gene flow and geographical isolation on genetic variation exhibited within and among sweet sorghum populations.The high level of gene flow observed in the present study had a role to bring a new allele within each sweet sorghum population. In addition this high level of gene flow resulted to increase the genetic diversity within the sweet sorghum population and reduce the diversity exihibited among populations. The private alleles observed among the studied accessions provides more evidence for novel alleles that can be used for further studies. These alleles uniquely found in a specific population, can be used as a source of desirable traits. The genetic variations observed so far in these accessions can be a basis to work on sugar content, drought resistance and other important agronomic traits. Since Ethiopia is the center of origin and diversity for sweet sorghum, more accessions should be collected and studied from all agro-ecologies. In addition, large number of markers should be employed to show the extent of molecular diversity in a better way.

## Supporting information

S1 Data Recorded molecular size of 91 sweet sorghum accessions that are clustered into 5 populations amplified using 13 SSR markers. (XLSX)
